# The Last Number

**Published:** 1869-03-01

**Authors:** 


### EDITORIAL.

### *The Last Number*

Of THE JOURNAL inadvertently went to press and was past praying for before it was noticed that the Editorial Department was not represented save by Book Notices. The highly valuable original matter offered to our readers in its pages cannot have escaped the special notice of our readers.

The notice of Dr. Phillip's book is marred by the omission of an "h" from "Ophthalmia." For "Trumbull," read "Turnbull." The proof was read by a non-professional. For sale by Walker & Son, McVicker's Theatre Building.

